# On the Evolving Biology of Language

**DOI:** 10.3389/fpsyg.2015.01796

**Published:** 2015-11-24

**Authors:** Dieter G. Hillert

**Affiliations:** ^1^School of Speech, Language, and Hearing Sciences, San Diego State UniversitySan Diego, CA, USA; ^2^School of Medicine, University of CaliforniaSan Diego, La Jolla, CA, USA

**Keywords:** *Homo erectus*, *ARHGAP11B*, *SRGAP2*, cultural evolution, biological disposition, brain development, language evolution, Neanderthals

## Introduction

Some language scientists defend an anti-Darwin account and believe in the saltational evolution of modern language. They emphasize that the language faculty emerged by a sudden mutation in the last 50–100 ky (e.g., Klein, 2000; Chomsky, 2012, 2015; Berwick et al., 2013). In contrast, others claim that modern language is the product of a gradual co-evolution of neurobiological and cultural-linguistic conditions, which took place since genus Pan was separated for good from the hominin lineage about 4–6 mya (e.g., Pinker and Bloom, 1990; Pinker, 1994; Deacon, 1997; Dor and Jablonka, 2001; Falk, 2004; Enfield and Levinson, 2006; Levinson and Jaisson, 2006; Christiansen and Chater, 2008; Atkinson, 2011; Dunn et al., 2011; Dediu and Levinson, 2014). New genetic evidence and their interpretation in context of fossil and artifact discoveries shed however light on this controversy. The data indicate that pre-modern language might have been already spoken by *Homo erectus*. Moreover, we conclude that the sister species of modern humans, Neanderthals and Denisovans, may have used language much like modern humans do (e.g., Dediu and Levinson, 2013).

To begin with, it is important to distinguish between the biology of modern language, that is, the *language-ready brain* and the availability of a *linguistic code*. For instance, a particular Homo species might have had a (pre-)modern language-ready brain but the language that was used at that time may have been different from modern language. Basic design features including combinatory, compositional as well as complex hierarchical structures are cultural products, which may have co-evolved along with genetic changes over a long period of time.

Modern language as we experience it today may require not only cortical wiring for language-specific operations but also a certain cortical mass to express thoughts and complex concepts. It would go beyond the present scope to discuss details but we assume here that the complex properties of modern language at all linguistic levels are from an evolutionary viewpoint mutually related to the principles of complex concept formations. An increase of the cranial capacity resulted in forming multi-modal memory systems. It seems thus plausible to assume that spoken language processing skills evolved alongside in order to communicate these sensory and episodic experiences.

The dramatic brain growth started with *Homo habilis*, which is sometimes considered to have an anagenetic relationship to the species *H. erectus* (Spoor et al., 2007). Again, sometimes *Homo heidelbergensis* has been classified as part of the *H. erectus* group by taking account of a broad range of individual differences (Lordkipanidze et al., 2013). The species *H. habilis* was around for 1 my, *H. erectus* for 1.6 my and *H. heidelbergensis* for 400 ky. As we will see below, the *H. erectus* epoch might have provided the opportunity for gradual biological adaptions resulting in refined language use. Recent genetic studies revealed factors, which may have significantly contributed to brain growth in human ancestors. Interestingly, these genetic factors seem to correspond to discrete cognitive stages in the human lineage.

## Genetic mutations for brain growth

The human brain, whose size tripled compared to genus Pan in a period of approximately 4 my, is not the result of a single mutation but involves multiple mutations. These genetic changes were dependent on each other and these changes were mutually dependent and favored by natural selection. Multiple genes are involved in the growth of the human brain (Fortna et al., 2004), but recent studies show that two different genes, *ARHGAP11B* and *SRGAP2*, seemed to have played a major role in cortical (re)organization in hominins. Brain growth for the evolution of modern language at all linguistic levels is crucial.

The gene *ARHGAP11B* was partially copied from *ARHGAP11A* after the hominin lineage split for good from genus Pan. As far as known, this copy exists only in modern humans, Neanderthals and Denisovans. In using an embryonal mouse model, Florio et al. (2015) showed that *ARHGAP11B* has the highest degree of radial glia-specific expressions. It promotes basal progenitor generation and proliferation, increases the cortical plate area and induces gyrification. *ARHGAP11B* can induce the folding of the developing mouse neocortex and may have contributed to the expansion of the human neocortex and their biological ancestors.

Also, the gene duplicates of *SRGAP2* significantly contributed to the expansion of the neocortex while the transition from Australopithecus to Homo took place. Dennis et al. (2012) found a copy-code mechanism, that is, in addition to the ancestral duplications at chromosome 1 further copies of *SRGAP2* were discovered. Thereby, the copies are not complete duplicates but are missing a small piece of the ancestral gene. The first duplications *SRGAP2A* and *SRGAP2B* were created ca. 3.2 mya and *SRGAP2C* was copied from *SRGAP2B* about 2.4 mya. Finally, the copy *SRGAP2D* appeared 900 kya, also a mutation of *SRGAP2B*. In particular *SRGAP2C* seemed to have accelerated cortical connectivity as indicated by mouse models. Charrier et al. (2012) found in knockdown mice that the ancestral *SRGAP2* will be mimicked by *SRGAP2C* and that neurons travel faster to the target areas as compared to neurons without the C variant. The inhibition of *SRGAP2* by human-specific paralogs certainly has played an important role in human brain development and thus presumably for the development of complex cognitive abilities. The occurrence of particular *SRGAP2* copies seems to correspond to some extent to certain epochs, in which new hominins with larger brains evolved and repopulated our world.

Australopithecus lived in Africa between 4 and 2 mya and had an average brain size of 450 cc, which is comparable to the size of chimpanzees. In this epoch, ca. 3.4 mya, the mutations *SRGAP2A* and *SRGAP2B* occurred. In particular *SRGAP2C*, a copy of *SRGAP2B*, significantly contributed, about 2.4 mya, to the growth of the human neocortex. The dating of the fossils classified as *H. habilis* (2.4–1.4 mya) and *H. erectus* (1.9–0.3 mya) corresponds to the occurrence of this genetic copy mechanism. On average, *H. habilis* had had a cranial capacity of 600 cc and *H. erectus* between 800 and 1000 cc. Finally, *SRGAP2D* appeared about 900 kya, which is as *SRGAP2C* a copy of *SRGAP2B*. The D-variant corresponds to the appearance of *H. heidelbergensis* or late *H. erectus* (200–600 kya). Typically, *H. erectus* is considered to be the direct ancestor of Neanderthals, Denisovans and modern humans. All three species co-existed and intermingled in Eurasia (e.g., Green et al., 2010; Meyer et al., 2012). Neanderthals lived throughout Europe and Middle East 28–200 kya, Denisovans fossils found in Siberia were dated to be 41 ky old and our species appeared in Africa about 200 kya and began to migrate to Eurasia ca. 60 kya. The brain volume of the Neanderthals was with 1 cc on average 250 cc larger than of modern humans. The Denisovans' brain size probably matched this range but because only one finger bone and two teeth were found (Krause et al., 2010), it is difficult to draw conclusions about their cranial capacity. In contrast to genus Pan, Neanderthals, Denisovans and modern humans obtained *ARHGAP11B* and the relevant *SRGAP2* copies.

Although, the factor brain volume alone does not inform about neural connectivity and the type of cortical circuits, we can certainly state that during a period of 3 my *SRGAP2* mutations played a major role in brain development. It is estimated that the adult brain of modern humans has ca. Twenty billion neocortical neurons and a single neuron has on average 7000 synaptic connections (Drachman, 2005; Herculano-Houzel, 2009). If the copy-code mechanism of *SRGAP2* was significant for the development of cognitive abilities and thus presumably also for language, the hypothesis of a sudden mutation, which brought about the biology disposition of language, seems not to be a convincing hypothesis. Instead, a non-expressed latent mutation in genus Australopithecus has been the precondition for further expressed mutations to promote brain volume and reorganization in the lineage of genus Homo.

The biological disposition for cognition and language seems thus to be the result of a gradual mutation, which may have begun already 3 mya. This hypothesis is certainly not a conclusive presumption. We do not know at which point in time the biological disposition of (pre-)modern language evolved, but in considering the reported genetic data on the neocortical evolution, it seems that basic principles supporting modern language were biologically disposed much earlier than previously assumed (Dediu and Levinson, 2013).

## From vocalization to spoken language

Along with the evolving biological disposition of language linguistic knowledge must have developed. Since genetic data, fossils and artifacts do not inform us directly about social and linguistic behavior, the following assumptions are certainly speculative. Recently, however, the timeline for stone artifacts has been pushed back by about 700 ky (Harmand et al., 2015). The stone tools from Lomekwi 3 (West Turkana, Kenya), which are considered to be part of the Oldowan culture, are dated to be 3 my old. The only hominin species known to have lived in this region around this time is a Kenyan variant of Australopithecus afarensis. This species had an approximate brain size of 430 cc, which is comparable to the brain size of genus Pan. Apparently, Australopithecus made already use of an elaborated hand-motor control, which might indicate a reorganization of cortical structures as compared to genus Pan (Stout et al., 2008; Stout, 2010; Stout and Chaminade, 2012). Accordingly, perhaps Australopithecus was already able to use non-symbolic, but referential vocalizations (possibly in combination with facial expressions and gestures) to display basic emotions and perceptions.

During the *H. erectus* epoch, Oldowan tools were refined and gradually replaced by Acheulean tools, which indicates that along with the increase of the cranial capacity cognitive skills co-evolved. With the emergence of *H. erectus* (brain volume of ca. 1000 cc), the cranial capacity consisted not only of more neurons but also of more interconnections between neurons and different cortical areas to support cognitive processing. The invention of a linguistic code for words and phrasal structures might have been happened during the *H. erectus* epoch. This time window permitted a continuous cultural development.

Here, we do not discuss how basic vocalizations converged into phonological units with iconic and symbolic meanings or how lexical units were combined to phrases and sentences with a specific word order to express more complex meanings. The cultural-linguistic process certainly co-evolved gradually, but we do not know how many cultural steps were involved before modern language was created (e.g., Boyd and Richerson, 1985; Evans and Levinson, 2009). The descendants of *H. erectus* are the sister species Neanderthals and Denisovans, and modern humans. Presumably, the neocortices of Neanderthals and Denisovans were biologically similar to modern humans. For instance, Neanderthals seem to have undergone the same selective sweep for the speech related gene *FOXP2* as modern humans in comparison to genus Pan (Enard et al., 2002; Krause et al., 2007; but see Coop et al., 2008) and similar results seem to apply for the genetic base of auditory capabilities (Martínez et al., 2004).

## Conclusions

The viewpoint that the language faculty emerged as a result of a sudden mutation 50–100 kya, seems not to be well supported. As partly discussed, the black box of the evolution of cognitive systems is not completely opaque anymore. Genetic data, fossils, and artifacts clearly inform us about a possible evolutionary path, which resulted in cortical rewiring and modern language processing as known to us today. From a methodological viewpoint it is essential to differentiate between the biological and cultural-linguistic evolution. Here, we believe that *H. erectus* and possibly its related (sub)species might have invented fundamental structures as found today in modern languages. In considering current genetic evidence about the cortical development in the hominin lineage, there is no reason not to believe that the known sister species Neanderthals and Denisovans (and at present unknown related species) used spoken language much like modern humans do.

### Conflict of interest statement

The author declares that the research was conducted in the absence of any commercial or financial relationships that could be construed as a potential conflict of interest.
